# Brief Report: Improving Early Infant Diagnosis Observations: Estimates of Timely HIV Testing and Mortality Among HIV-Exposed Infants

**DOI:** 10.1097/QAI.0000000000002263

**Published:** 2020-01-06

**Authors:** Karen Webb, Vivian Chitiyo, Nyikadzino Mahachi, Solomon Huruva Mukungunugwa, Angela Mushavi, Simukai Zizhou, Barbara Engelsmann, Rashida Abbas Ferrand, Melissa Neuman, Wendy Hartogensis, Elvin Geng

**Affiliations:** a Organization for Public Health Interventions and Development, Harare, Zimbabwe;; b London School of Hygiene and Tropical Medicine, London, United Kingdom;; c Ministry of Health and Child Care, National PMTCT Program, Harare, Zimbabwe;; d Ministry of Health and Child Care, Mashonaland East Province, Zimbabwe; and; e University of California, San Francisco, CA.

**Keywords:** HIV-exposed infants, early infant diagnosis, loss to follow-up, prevention of mother-to-child HIV transmission, retention

## Abstract

**Background:**

Improving efforts toward elimination of mother-to-child transmission of HIV requires timely early infant diagnosis (EID) among all HIV-exposed infants, but the occurrence of timely EID and infant survival may be underascertained in routine, facility-bound program data.

**Methods:**

From March 2015 to May 2015, we traced a random sample of HIV-positive mother and HIV-exposed infant pairs lost to follow-up for EID in facility registers in Zimbabwe. We incorporated updated information into weighted survival analyses to estimate incidence of EID and death. Reasons for no EID were surveyed from caregivers.

**Results:**

Among 2651 HIV-positive women attending antenatal care, 1823 (68.8%) infants had no documented EID by 3 months of age. Among a random sample of 643 (35.3%) HIV-exposed infants lost to follow-up for EID, vital status was ascertained among 371 (57.7%) and updated care status obtained from 256 (39.8%) mothers traced. Among all HIV-infected mother–HIV-exposed infant pairs, weighted estimates found cumulative incidence of infant death by 90 days of 3.9% (95% confidence interval: 3.4% to 4.4%). Cumulative incidence of timely EID with death as a competing risk was 60%. The most frequently cited reasons for failure to uptake EID were “my child died” and “I didn't know I should have my child tested.”

**Conclusions:**

Our findings indicate uptake of timely EID among HIV-exposed infants is underestimated in routine health information systems. High, early mortality among HIV-exposed infants underscores the need to more effectively identify HIV-positive mother–HIV exposed infant pairs at high risk of adverse outcomes and loss to follow-up for enhanced interventions.

## INTRODUCTION

The timeliness of early infant diagnosis (EID)—HIV testing of exposed infants 6–8 weeks after birth—and proportion of infants testing positive are cardinal indicators of prevention of mother-to-child transmission (PMTCT) program success.^1^ However, in routine program settings, completion of EID is assessed at individual facilities, from information across several paper-based registers, which may be incomplete and/or inaccurate.^2,3^

New approaches are needed to improve confidence in estimates of EID completion and survival of HIV-exposed infants to guide on-going quality improvement and inform national modeling estimates. These become increasingly important as countries such as Zimbabwe, with an HIV prevalence of 16.0% among women,^4^ seek to validate elimination of mother-to-child transmission.^5^

In this study, we randomly sampled lost-to-follow-up (LTFU) HIV-positive mother, HIV-exposed baby pairs (MB), pairs from routine health facility-based data. We actively traced MB pairs in the community to assess infant and maternal survival and EID uptake. Finally, we incorporated these findings to correct estimates of HIV-exposed infant mortality and timely EID in the entire clinic population of MB pairs using a probability weight. Although this sampling-based approach has been used in clinic-based cohorts of adult patients in HIV treatment programs and found to alter estimates of retention and mortality,^6,7^ our study represents the first time the approach has been used in PMTCT programs. We reflect on key lessons from our research on program progress and persisting gaps for strengthening EID coverage on the path to elimination of MTCT in Zimbabwe.

## METHODS

### Study Design

We defined LTFU as no documented sample taken for EID polymerase chain reaction testing among infants of HIV-positive antenatal care (ANC) patients within 90 days of delivery. At the time of study, Zimbabwe HIV care and treatment guidelines recommended HIV testing of all exposed children 6 weeks postnatal.^8^ Our sample included all women with HIV who accessed ANC at public sector health facilities in Mashonaland East Province (referral sites excluded), using a multistage clustered survey sampling approach.

#### Phase I: Register Tracing

We selected 5 clinics from each of the 9 districts within Mashonaland East Province based on probability proportional to size, using program data on HIV-positive women accessing ANC over the previous year. In the absence of interfacility-linked electronic patient monitoring systems, we used a combination of individual identifiers including clinic-allocated PMTCT number, patient name, and date of birth to trace each identified HIV-positive pregnant woman in ANC and her HIV-exposed infant through 5 paper-based registers (ANC, delivery, HIV-exposed infants, clinic registers, HIV infant diagnosis, and EID lab request form booklets) to determine LTFU for EID status. We targeted a random sample of at least 10% of lost MB pairs based on practical considerations regarding limitations of completeness and accuracy of patient information in multiple paper-based registers^9^ and available resources to intensively trace those identified as LTFU for EID over the study timeframe. Based on known challenges in tracing defaulters from HIV care and treatment,^10–12^ we over-sampled by 100% to ensure we met our target sample.

#### Phase II: Tracing of LTFU MB Pairs

In the second stage of sampling, a random sample of patients identified as lacking documented EID in Stage 1 was selected for active tracing to determine outcomes through direct patient interviews. We trained MOHCC village health workers as ascertainers due to their familiarity with the surrounding community and existing role to support health facilities with defaulter tracing.^13,14^

Village health workers were assigned selected LTFU patients residing within their geographical catchment area for tracing. If the individual was unable to be located, mother and infant vital status was obtained from informants (friends, neighbors, or relatives); however, to maintain confidentiality, informants were not asked any specific questions related to HIV services. Successfully traced and consenting mothers were interviewed using a standardized questionnaire to ascertain survival outcomes, EID status and timing, and reasons for failure to obtain EID or for self-transfer of care to another facility. Analyses were conducted using Stata v.13.1. This study was approved by the Medical Research Council of Zimbabwe (MRCZ/A/1844) and MOHCC authorities.

### Analyses

#### EID Estimates

First, we estimated EID incidence based on clinic registry data only, using facility weights inverse to the probability of clinic selection to yield an estimate of EID completion based purely on paper registers kept at the facilities. Facility and individual factors on documented completion of EID were explored using Poisson regression with robust standard errors adjusted for confounders to estimate risk ratios (RRs).^15,16^

Second, we generated corrected estimates of EID completion using data from clinic registries as well as data ascertained through interviews with a random sample of MB pairs missing EID in facility-based registers.^7^ Deaths before EID (including fetal deaths) were considered as competing risks. To obtain an estimate corrected for outcomes not captured in the facility registers, we used additional sampling weights inverse to the probability of being successfully sought to represent all LTFU HIV-infected pregnant women at each clinic.

Among women with no documented EID in facility registers who were traced and interviewed, reasons for silent transfer to a different clinic from ANC for EID and for not bringing HIV-exposed child in for HIV testing (no EID) were grouped into 4 categories informed by a socioecological framework^17,18^: structural; clinic-based; psychosocial or patient-related; or medical factors.

#### Mortality Estimates

For the weighted mortality estimate, the traced patients contributed time from the delivery date to the date of the patient interview or the date of the death of the infant at any time prior (ie, all deaths including those later than 90 days were included in the mortality estimate); patients with documented EID contributed time from the date of delivery to the date of the 6-week postnatal visit, which is the latest date at which we have confirmed vital status for those infants. Kaplan–Meier methods were used to estimate mortality.

## RESULTS

### Phase I: Facility Register Data

Among 18,065 women registered for ANC between April 2012 and May 2013, 2651 (14.7%) were HIV positive and 31.2% [95% confidence interval (CI): 29.5% to 33.0%] had documented uptake of EID for their infant within 3 months of delivery in clinic registers. After adjustment for register information and site characteristics, factors associated with documented EID completion included earlier gestational age at presentation (RR: 0.97 per 2 weeks; 95% CI: 0.95 to 0.99; *P* = 0.013), later calendar time of ANC presentation (RR: 1.04 per 30 days; 95% CI: 1.02 to 1.06, *P* = 0.011), and smaller site volume (RR: 1.85 1-200 ANC patient volume, 1001-1500 volume referent; 95% CI: 1.44 to 2.38, *P* < 0.001).

### Phase II: Community Tracing LTFU

**FIGURE 1.**
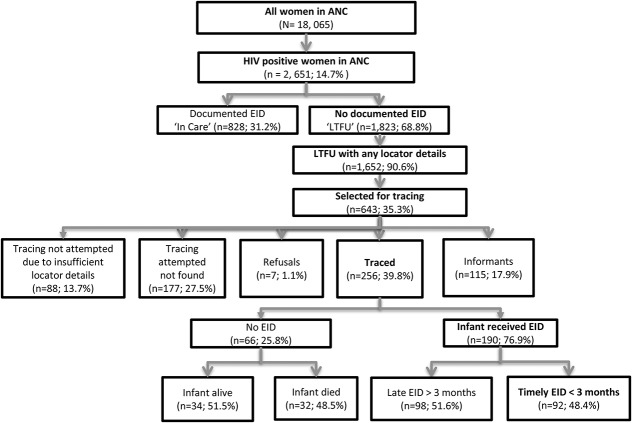
Flowchart of outcomes in the study population (N = 18,065).

Among 1652 mother–baby pairs identified as LTFU with any documented locator information, a random sample of 643 (38.9%) was selected for community tracing between March 2015 and May 2015. In 371/643 (57.7%), updated vital or EID status information was obtained (22.5%; 371/1652 of the total LTFU sample). The primary reason for failure to locate clients was insufficient location information. Among 371 successfully traced patients, 256 (69.0% of located) mothers were interviewed directly on infant vital status and EID uptake, and for the remaining 115 (31.0%), informants were interviewed regarding MB pair survival outcomes (not HIV-related) (Fig. 1). Among the 371 mother–baby pairs for whom vital status outcomes were determined, 66 infants (17.8%; 95% CI: 14.0% to 22.1%) and 18 mothers (4.9%; 95% CI: 2.9% to 7.6%) were found to be deceased.

**FIGURE 2.**
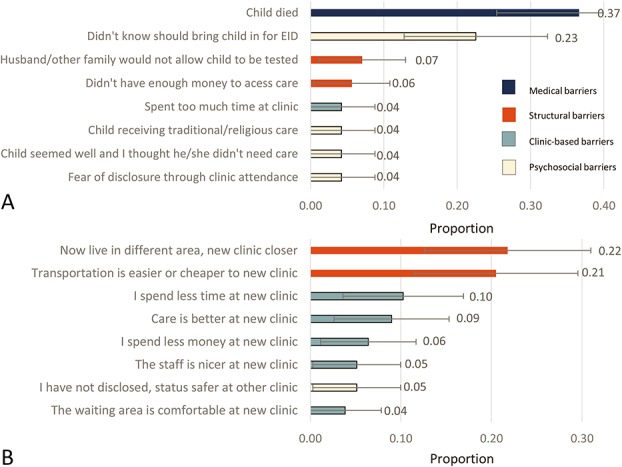
Prevalence of patient-reported reasons for no Early Infant Diagnosis (EID) (N = 71) (A) and switching site of EID from ANC care (N = 78) (B). Structural barriers stem from material conditions of life in resource-limited settings (eg, transportation cost and availability, family conflict, or not enough money). Psychosocial barriers are related to knowledge, beliefs, or attitudes of the patients in the given social setting (eg, “didn't know” child should be tested, fear of disclosure, or preference for spiritual healing). Clinic-based barriers are related to delivery processes at a clinic site (eg, long waiting times, healthcare worker friendliness, and quality of care). Medical barriers related to health status (such as infant death or mother too sick to the bring child to the clinic).

Most mothers interviewed (190/256; 74.2%; 95% CI: 68.4% to 79.5%) reported their infant had received HIV testing; although fewer than half received EID testing before 3 months of age (92/190; 48.4%; 95% CI: 41.1% to 55.8%). Our corrected estimate following tracing resulted in a cumulative incidence of EID with death as a competing risk of 60.0% (95% CI: 58.7% to 61.3%). We estimated a cumulative incidence of mortality among HIV-exposed infants at 3 months of 3.9% (95% CI: 3.4% to 4.4%) and at 1 year of 7.7% (95% CI: 4.7% to 13.5%). Among the 66 infants with no EID at any time, the most frequently cited reason for failure to have EID was “my child died” (36.6%; 95% CI: 25.8% to 49.0%), Figure 2A. Among infants with timing of death ascertained, most did not survive to the age of recommended EID testing (6 weeks) (26/42; 61.9%). Among mothers of living infants, “I didn't know I should have my child tested” was the most frequently cited reason for no EID (16/45; 35.6%). Relocation to a different area (21.8%; 95% CI: 13.2% to 32.6%) and transport being easier/cheaper at new clinic (20.5%; 95% CI: 12.2% to 31.1%) were the most commonly reported reasons for silent transfer (Fig. 2B).

## DISCUSSION

In a representative sample of Mashonaland East Province of Zimbabwe, after tracing a sample of MB pairs identified as LTFU for EID, our corrected estimate of EID almost doubled (from 31.2% to 60.0%). These findings underscore the risk of equating LTFU of MB pairs in health information systems with disengagement from care where such systems do not facilitate electronic, longitudinal tracing of service uptake within and between health facilities.^19^ MOHCC is currently expanding efforts to strengthen facility-based documentation, retention monitoring, and longitudinal outcome reporting through use of the Mother-Baby Pair Register to track longitudinal outcomes of MB Pairs, together with appointment diary systems.^20^ As Zimbabwe transitions toward electronic health record systems, we indicate the value of sampling-based methods for improving accuracy of EID coverage estimates and informing current progress toward EMTCT validation.^21^

We found high mortality among HIV-exposed infants, with “my child died” being the most frequently cited reason for no EID testing. Passive facility-based monitoring may underestimate true mortality by up to 80%,^22^ and under-reporting of infant mortality in high HIV burden settings is acknowledged to bias child mortality estimates downward.^23,24^ Our findings reinforce the need to act early among HIV-positive mother–exposed infant pairs at high risk of defaulting or adverse clinical outcomes with enhanced PMTCT interventions.^25,26^ Subsequent MOHCC guidelines recommending prioritization of viral load monitoring for pregnant and lactating mothers, birth testing for high-risk infants,^20,27^ and case-based surveillance of infants testing HIV positive^20,28^ are intended strengthen program evidence and action among high-risk MB pairs. Our findings emphasize that monitoring of facility-level implementation fidelity, data quality, and robust analysis of resulting data will be central to realizing the benefit of such efforts for improved PMTCT program strategies and impact.

Finally, we not only improved our understanding of “true EID” rates but also developed evidence on factors influencing timely infant HIV testing for informing quality improvement. At patient-level, the predominance of psychosocial reasons (“I didn't know”) for failure to uptake EID among mothers of living infants emphasizes need to provide information about the importance of timely infant HIV testing during the initial engagement in ANC and continued emphasis at every subsequent visit. Reasons for silent transfer are consistent with studies of adult ART retention and highlight the role of structural^29^ and clinic-based factors^30^ for optimizing retention in care along the PMTCT cascade. Findings have guided PMTCT program planning and actions including strengthening of patient education and problem solving, appointment monitoring, active follow-up, and outcome documentation in Zimbabwe and other settings.^20,31–33^

### Limitations

Although oversampling enabled achievement of our targeted sample of at least 10% of MB pairs LTFU for EID (we traced 22%), our findings highlight need to strengthen routine documentation and other forms of observational data to ascertain uptake of services and mortality.^34^ We acknowledge nonresponse due to incomplete tracing details may introduce some uncertainty in the resulting estimates. However, our findings of lower LTFU and higher mortality after tracing are concordant with larger, more robust LTFU studies.^35^ In the absence of effective interfacility electronic patient-monitoring systems, we demonstrate the value of leveraging routine observational data in “real-life” program settings for improving accuracy of estimates, identifying bottlenecks and guiding programmatic actions at the local level.^34,36,37^

## CONCLUSIONS

Our findings suggest that routinely reported EID completion in public health information systems may be substantially underestimated. Accurate determination of mother–infant pair outcomes in PMTCT programs is complicated by resource-constrained health information systems that involve multiple paper-based registers, lack of unique identifiers, and challenges with completeness and accuracy of information recorded. We demonstrate the value of sampling-based approaches in pediatric HIV research for providing important, context-based evidence for policy and programs.
